# Lymphoepithelial Cyst of the Salivary Gland in a Small Ruminant Lentivirus-Positive Goat

**DOI:** 10.3390/ani10091545

**Published:** 2020-09-01

**Authors:** Izabella Dolka, Marek Tomaszewski, Daria Wola, Michał Czopowicz, Jarosław Kaba

**Affiliations:** 1 Department of Pathology and Veterinary Diagnostics, Institute of Veterinary Medicine, Warsaw University of Life Sciences, Nowoursynowska 159c, 02-776 Warsaw, Poland; 2 The Scientific Society of Veterinary Medicine Students, Institute of Veterinary Medicine, Warsaw University of Life Sciences, Nowoursynowska 159, 02-787 Warsaw, Poland; ar.szewski@gmail.com (M.T.); daria.wola@onet.pl (D.W.); 3 Division of Veterinary Epidemiology and Economics, Institute of Veterinary Medicine, Warsaw University of Life Sciences, Nowoursynowska 159c, 02-776 Warsaw, Poland; michal_czopowicz@sggw.edu.pl (M.C.); jaroslaw_kaba@sggw.edu.pl (J.K.)

**Keywords:** lymphoepithelial cyst, LEC, salivary gland, goat, CAE, SRLV, histopathology, immunohistochemistry

## Abstract

**Simple Summary:**

This study presents the first case of a lymphoepithelial cyst (LEC) adjacent to the salivary gland in a goat seropositive for the small ruminant lentivirus (SRLV). Immunohistochemistry detected the antigen of caprine arthritis-encephalitis virus (CAEV) in the LEC, salivary gland, and lung tissue. In human patients, a LEC of the major salivary gland is an uncommon benign lesion and may be the early clinical manifestation of human immunodeficiency virus (HIV) infection. The present report provides useful information on the comparative aspect of LEC in an animal infected with CAEV, one of the SRLVs usually associated with chronic infection. Although many hypothetic theories were proposed, the etiopathogenesis of LEC is still debated. In this study, we present the histopathological description of LEC, supported by histochemistry and immunohistochemistry.

**Abstract:**

The lymphoepithelial cyst (LEC) of the major salivary gland is a rare lesion described in medical literature. It is found in human immunodeficiency virus (HIV)-infected patients and considered an early manifestation of this infection. Despite the variety of theories, the origin of this lesion remains controversial. No veterinary studies on LEC have been published so far. This study is the first-ever that aims to describe histopathological, histochemical, and immunohistochemical features of a LEC located adjacent to the salivary gland of a goat. The goat proved seropositive for the small ruminant lentivirus, showed clinical signs of caprine arthritis-encephalitis, and had caprine arthritis-encephalitis virus (CAEV)-infected cells in the lung. The histopathology revealed a cystic lesion lined mainly with squamous epithelium surrounded by a lymphoid component, containing a mucus-negative material and a few nonbirefringent structures corresponding to amylase crystalloids. Using immunohistochemistry, CAEV-positive cells were detected in macrophages, LEC epithelial cells, and the salivary gland. The B cells were mainly in the germinal centres, the intraepithelial lymphocytes expressed CD3 and Bcl-2, and the proliferative activity was low. This study showed that LEC had many similar histological and immunohistochemical features to those seen in humans. However, further studies are required in this respect.

## 1. Introduction

The lymphoepithelial cyst (LEC) is a rare benign lesion, uncommonly diagnosed in veterinary and human medicine. It has mainly been discovered in the human head-and-neck region, such as the oral cavity and salivary glands (more commonly, the parotid and, rarely, the submandibular gland), most often in the lateral cervical area just below the angle of the mandible, including the lymph nodes. It rarely affects the thyroid gland, lungs, and pancreas [1].

The LEC of the salivary glands may be single or multiple, uni- or multilocular, or unilateral or bilateral, and its size varies from a few millimetres to centimetres (range 0.5–5 cm). It appears almost equally in both sexes as a soft, painless mass that grows slowly and may cause facial asymmetry. A complete surgical removal remains the treatment of choice and generally has a good prognosis. Human immunodeficiency virus (HIV)-related LEC is more prone to the transformation into B-cell lymphoma [2,3].

The LEC was first described in 1885 as a benign lymphocytic infiltration and an enlargement of the salivary and lacrimal glands, but the AIDS-related involvement of lymph nodes of the salivary glands was first reported in 1985 [4,5]. The LEC has been widely detected as a common cause of an enlarged parotid gland in patients infected with human immunodeficiency virus (HIV). The incidence of the salivary LEC in HIV-infected patients ranges between 3% and 6%. It is typically observed in middle-aged patients of both sexes and is usually large, bilateral, and multiloculated. Its presence should be considered as a hallmark of the early phases of HIV infection, whereas it is rarely observed in patients with an advanced stage of HIV infection [6,7]. It has been considered as nearly pathognomonic for HIV. In addition, its decreased incidence upon an antiretroviral treatment used as the first-line therapy has proved that this “linking” is not purely coincidental [1].

Despite the growing interest in LEC due to its relation to HIV infection, its etiopathogenesis remains controversial, especially due to its occurrence in non-HIV patients [8].

The diagnosis of HIV-associated LEC is usually established by the medical history, clinical appearance, blood tests for HIV, preoperative imaging, cytology (fine-needle aspiration), and histopathology (haematoxylin-eosin staining—HE) [2,3,9]. There are only a few reports that confirmed HIV-associated LEC via HIV-1 p24 immunohistochemical stainings [9,10,11,12]. Sekikawa et al. [9] suggested that, in HIV-patients with suspected LEC, this staining could help in the differential diagnosis. However, it is not offered in the standard practice of many laboratories.

Caprine arthritis-encephalitis (CAE) is a contagious disease of goats caused by the caprine arthritis-encephalitis virus (CAEV) classified as a small ruminant lentivirus (SRLV). SRLV belongs to the *Retroviridae* family closely related to HIV. CAE is a chronic progressive disease manifesting as chronic non-purulent arthritis, wasting, less often indurative mastitis, and interstitial pneumonia [13,14]. The presence of LEC linked to SRLV-positive animals is unknown, and the pathological features of LEC have not been fully characterized so far. In this study, the histopathological and immunohistochemical characteristics of LEC in the salivary gland are described in a SRLV-positive dairy goat. The comparative aspect to its human counterpart is also discussed.

## 2. Materials and Methods

### 2.1. Case Presentation

A 5-year-old Polish White Improved dairy goat was seropositive for SRLV using ELISA (ID Screen MVV-CAEV Indirect Screening test, ID.vet Innovative Diagnostics, Grabels, France). The goat came from a herd with a long history of CAEV infection (with seroprevalence exceeding 90%) and the severity of pathological findings varying considerably among individual goats. The present goat showed clinical signs indicative of CAE—weight loss, marked dyspnoea, cyanosis of the oral mucosa, and the swelling of the carpal joints, suggesting arthritis. The animal was euthanized at the owner’s request and submitted for necropsy to the Department of Pathology and Veterinary Diagnostics, Warsaw University of Life Sciences (WULS). Additionally, other animals from the herd demonstrated clinical symptoms (lameness, emaciation, and interstitial mastitis) and macro- and microscopical lesions consistent with CAE.

Grossly, the body condition of the goat was poor (2 on the 5-point scale) [15]. Skin abrasions were also noted on the dorsal surfaces of the carpal joints and the sternum. The joint capsules were thickened and contained quite a small amount of synovial fluid. The surface of the articular cartilage was smooth. The mammary gland seemed to be empty. Mammary gland lymph nodes were congested. Pneumonia was the most evident lesion in the present case. The lungs were diffusely enlarged, firm, and congested, with consolidated cranioventral lobes and scattered grey-pink foci. The lungs did not collapse, and the hydrostatic test of the pulmonary samples showed flotation. Moreover, mainly the left cranial lobe showed fibrinous exudate on the cross-section, and its pleura was covered with fibrin deposits. Some fibrous adhesions extended onto the parietal pleura and pericardial sac. The tracheobronchial lymph nodes were enlarged and firm. The thoracic cavity contained blood-tinged and slightly cloudy fluid. Other pathological findings included pericarditis, splenic congestion, small duodenal haemorrhages, and renal pelvis calculi. Additionally, an oval, circumscribed mass of a cyst-like structure (about 2 cm in diameter) was found on the cross-section close to the right mandibular angle. The cyst was unilocular, surrounded by a thin wall (about 0.5 cm), and filled with a soft, pasty cream-coloured material (“muddy” appearance). The brain was not examined.

### 2.2. Histopathology

Tissue samples from the cyst-like lesion of interest, and from the lungs, spleen, heart, and duodenum, were fixed in 10% buffered formalin, routinely processed, and embedded in paraffin. The 4-μm-thick sections were stained with HE. Furthermore, the cystic mass with adjacent parenchyma was examined using histochemical stains, such as periodic acid Schiff (PAS), PAS with alcian blue (PAS-AB), alcian blue at pH 2.5, PAS-diastase (PAS/D), mucicarmine, Papanicolaou (Pap), and May-Grünwald-Giemsa (MGG). All samples were reviewed using an Olympus BX43 microscope (Olympus Corporation, Tokyo, Japan).

### 2.3. Immunohistochemistry

Immunohistochemistry (IHC) of the cystic lesion was performed using primary antibodies (Table 1). Moreover, the lung specimens were analysed for the presence of a CAEV p28 antigen.

After dewaxing in xylene and rehydration in alcohols, the slides were microwaved in 0.02-M citrate buffer, pH 6.0, or in TRIS-EDTA, pH 9.0, at 600 W for 15 minutes and then treated with a 3% perhydrol solution for 15 minutes. The sections were incubated with the primary antibody for 1 hour at room temperature (RT) in a humid chamber; however, with anti-CAEV antibody overnight at 4 °C. After washing in Tris-buffered saline, they were incubated with a biotinylated secondary antibody (EnVision™ detection system, Peroxidase/DAB (3,3’-diaminobenzidine), Rabbit/Mouse, HRP, Dako, Agilent Tech., Santa Clara, CA, USA) and then visualized with 3,3’-diaminobenzidine (DAB) from Dako, Agilent Tech., Santa Clara, CA, USA. The sections were counterstained with Erlich’s haematoxylin and mounted using the DPX medium (Sigma-Aldrich Co, St. Louis, MO, USA). Negative controls were performed by omitting the primary antibody. Moreover, an isotypical monoclonal IgG was used as a negative control, replacing the anti-CAEV antibody. Tissues with known positive reactivity from a dog and goat (lungs, skin, mammary gland, salivary gland, spleen, tonsil, lymph nodes, and uterus) were used as positive controls.

## 3. Results

### 3.1. Histopathological Results

**Figure 1:**
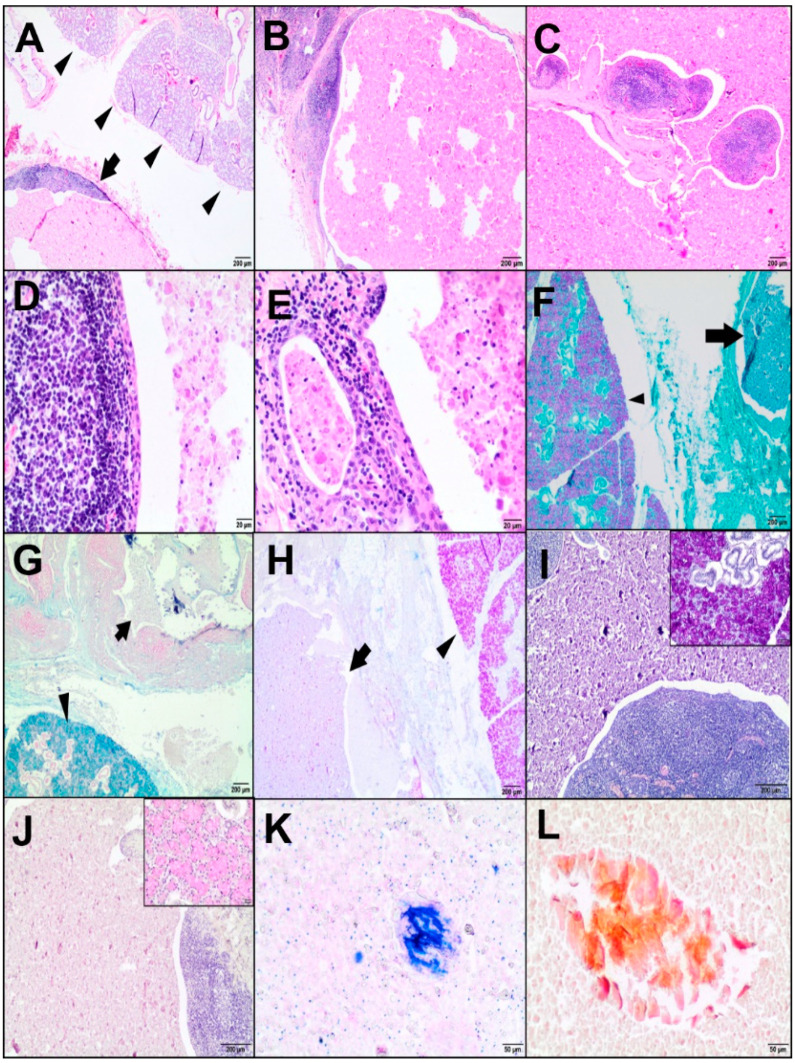
Histopathological (**A**–**E**) and histochemical examinations (**F**–**L**) of the lymphoepithelial cyst (LEC). (**A**) The LEC (arrow) close to the salivary gland (arrowheads). Haematoxylin-eosin staining (HE), 20×; scale bar = 200 µm. (**B**) The cystic cavity with eosinophilic material surrounded by lymphoid tissue. HE, 20×; scale bar = 200 µm. (**C**) Thin fibrous bands containing subepithelial lymphoid tissue. HE, 20×; scale bar = 200 µm. (**D**) The cyst lined by squamous epithelium: small lymphoid follicles with a germinal centre beneath the lining of the epithelium. HE, 200×; scale bar = 20 µm. (**E**) A ductal structure lined by cuboidal epithelium located beneath a cyst. HE, 200×; scale bar = 20 µm. (**F**) Mucous acini positive for periodic acid Schiff (PAS) (arrowhead, magenta colour). PAS-negative content of the cyst (arrow), 20×; scale bar = 200 µm. (**G**) Mucous acini rich in acid mucopolysaccharides positive for alcian blue (AB) (arrowhead, blue). AB-negative content of the cyst (arrow), 20×; scale bar = 200 µm. (**H**) PAS-AB-stained neutral mucins in the salivary glands (arrowhead, magenta). PAS-AB-negative cystic content (arrow), 20×; scale bar = 200 µm. (**I**) The cystic content negative for PAS-diastase (PAS/D), 40×; scale bar = 200 µm. Inset: Acinar cells magenta-coloured in PAS/D, 100×; scale bar = 50 µm. (**J**) The cystic content negative for mucicarmine, 40×; scale bar = 200 µm. Inset: Acinar cells positive for mucicarmine (pink), 200×; scale bar = 20 µm. (**K**) Crystalloids (blue). May-Grünwald-Giemsa (MGG), 100×; scale bar = 50 µm. (**L**) Orangeophilic-stained crystalloids in the cystic cavity. Pap, 100×; scale bar = 50 µm.

Histopathology of the mass in the right angle region of the mandible revealed a cyst located adjacent to the salivary gland. The cyst was lined by squamous epithelium with underneath lymphoid tissue. In some areas, the epithelium appeared to be transitional or low cuboidal, and intraepithelial lymphocytes were noted. The cyst was surrounded by a rim of connective tissue forming a pseudocapsule. It was separated by thin fibrous bands of well-vascularized fibroconnective tissue stroma containing subepithelial lymphoid tissues with a few small lymphoid follicles, some of which had relatively small germinal centres. Normal lymph node structures were difficult to determine. Focally within the lymphoid component, there were single ductal structures lined by flat to cuboidal epithelium (Figure 1A–E).

The cystic cavity contained a granular, eosinophilic (proteinaceous) material mixed with desquamated epithelial cells, lymphocytes, macrophages, and single glassy, nonbirefringent geometric structures (7.8–176.5 μm in diameter), which resembled amylase crystalloids. The content was negative for PAS, PAS-AB, AB, and PAS/D mucicarmine, but some crystalloids stained orange with Pap and blue with MGG (Figure 1F–L). The salivary glandular tissue adjacent to the cystic lesion consisted of serous, mucous, mixed acini with serous demilunes, and striated ducts composed of acidophilic cuboidal/columnar cells. The acini stained magenta with PAS and PAS-AB indicated neutral mucins. In the PAS-AB staining, the contents of acid mucins stained bright blue in the seromucous endpieces. The mucous acinar cells showed the content of acidic mucopolysaccharides and were blue in AB. Moreover, they were positive for mucicarmine and PAS/D. The above findings were related to the submandibular gland.

The areas of interstitial pneumonia and fibrinopurulent bronchopneumonia were noted in the histological examination. The interstitial pneumonia was characterised by mild-to-moderate hyperplasia of lymphoid tissue in the alveolar septa, around the bronchi and arteries, pneumocyte and smooth muscle hyperplasia, the proliferation of connective tissue and accumulation of mononuclear cells (mainly lymphocytes and macrophages), the presence of macrophages, and an eosinophilic mass in some alveoli. However, lung samples also demonstrated areas of bacterial pneumonia superimposed on viral pneumonia. Alveoli and bronchiole were filled with neutrophils, macrophages, fibrin, and necrotic cellular debris. These findings were consistent with fibrinopurulent bronchopneumonia accompanied by fibrinous pleuritis. In addition, moderate lymphoid follicle hyperplasia and depletion of the splenic white pulp, mild lymphocytic myocarditis, focal fibrosis scattered sarcocysts in the myocardium, and chronic duodenitis were observed.

### 3.2. Immunohistochemical Results

**Figure 2:**
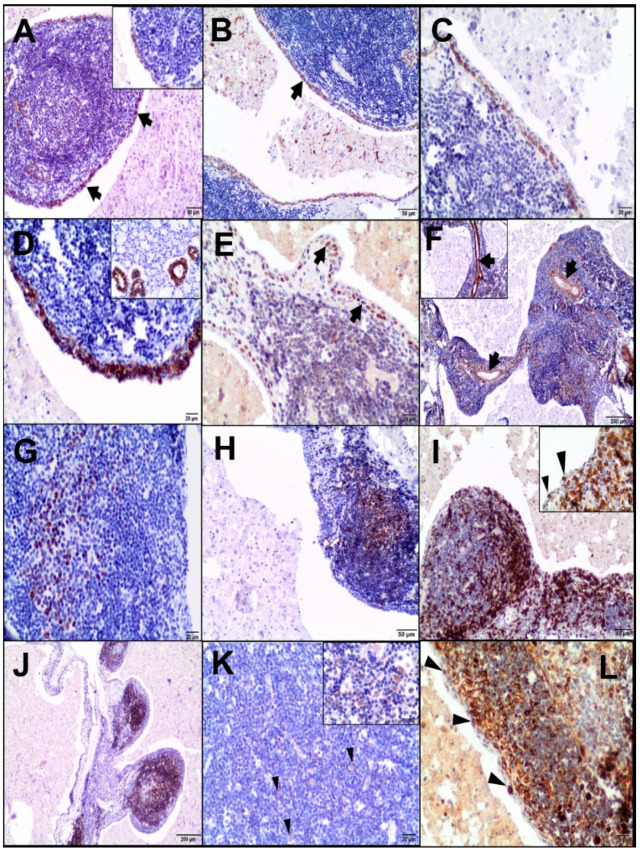
Immunohistochemical examination of the LEC. (**A**) Pan-CK-stained cytoplasm of the epithelial cells (arrows, inset, 400×, scale bar = 20 µm). Some pan-CK-positive reticulum cells in the background of the lymphoid tissue, 100×; scale bar = 50 µm. (**B**) Basal epithelial cells of the cyst positive for the squamous differentiation marker (CK5/6), 100×; scale bar = 50 µm. (**C**) CK14 expression in the basal cells of the squamous epithelium, 200×; scale bar = 20 µm. (**D**) CK19 stained the luminal epithelial cells, 200×; scale bar = 20 µm. Inset: CK 19 expressed in ductal cells but absent in acinar cells of the salivary gland, 200×; scale bar = 20 µm. (**E**) p63 expressed in basal cells of the squamous epithelium (arrows), 200×; scale bar = 20 µm. (**F**) Vim stained connective tissue stroma and the blood vessel wall (arrows), 40×; scale bar = 200 µm. Inset: αSMA-positive blood vessels, 100×; scale bar = 50 µm. (**G**) Nuclear positivity for Ki-67 in the lymphoid follicles, 200×; scale bar = 20 µm. (**H**) PCNA-positive cells in the germinal centre of the lymphoid follicles, 100×; scale bar = 50 µm. (**I**) CD3-positive lymphocytes mainly in the peripheral region of the lymphoid follicles and interfollicular regions. 100×; scale bar = 50 µm. Inset: CD3-positive intraepithelial lymphocytes (arrowheads), 400×; scale bar = 20 µm. (**J**) CD79α expressed in the marginal zone and less in the germinal centres, 40×; scale bar = 200 µm. (**K**) A few CD68-positive macrophages (arrowheads), 200×; scale bar = 20 µm. Inset: CD68 indicated by the cytoplasmic staining reaction, 400×; scale bar = 20 µm. (**L**) Bcl-2 staining (mostly cytoplasmic and nuclear) mainly in the marginal zone of the lymphoid follicles and a few intraepithelial lymphocytes (arrowheads), 200×; scale bar = 20 µm.

Immunohistochemistry for cytokeratins (CKs) highlighted the epithelial component of the cyst (the luminal layer, basal layer, and desquamated cells) (Figure 2A–D). 

**Figure 3:**
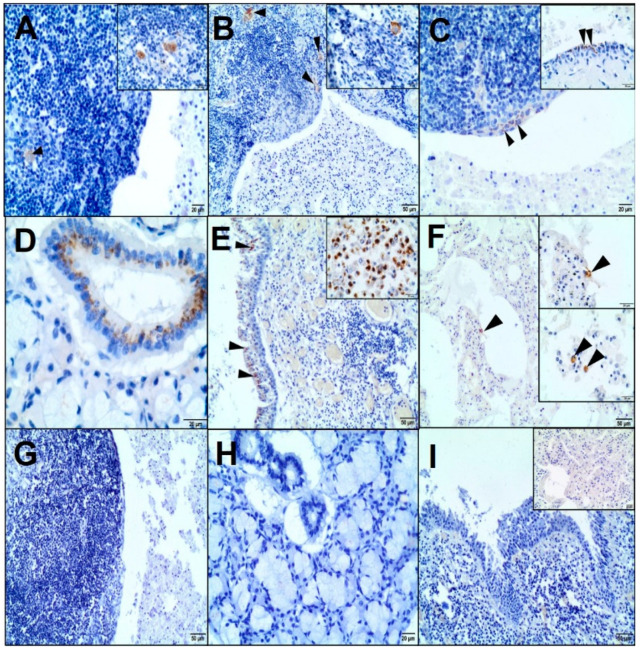
Immunohistochemical examination of the caprine arthritis-encephalitis virus (CAEV) in the LEC, salivary gland, and lungs. (**A**) The positive staining of CAEV (cytoplasmic) in macrophages and macrophage-like cells in the lymphoid follicles (arrowhead), 200×; scale bar = 20 µm. Inset, 400×; scale bar = 20 µm. (**B**) Additionally, in the peripheral lymphoid region (arrowheads), 100×; scale bar = 50 µm. Inset, 400×; scale bar = 20 µm. (**C**) The cytoplasm of the epithelial cells lining the cyst slightly immunostained with CAEV (arrowheads), 200×; scale bar = 20 µm. Inset, 400×; scale bar = 20 µm. (**D**) The immunostaining of CAEV (cytoplasmic, in the perinuclear region) in the epithelial cells of the salivary duct, 400×; scale bar = 20 µm. (**E**) CAEV-positive bronchial epithelial cells (cytoplasmic, perinuclear staining; arrowheads), 100×; scale bar = 50 µm. Inset: CAEV-positive macrophages in the peribronchial inflammatory cell infiltration, 400×; scale bar = 20 µm. (**F**) A few CAEV-positive macrophages scattered in the interstitial tissue and alveolar lumen, 100×; scale bar = 50 µm. Inset 400×; scale bar = 20 µm. (**G**–**I**) No specific staining was observed in the negative controls. (**G**) LEC, 100×; scale bar = 50 µm. (**H**) Salivary gland, 200×; scale bar = 20 µm. (**I**) Lungs, 100×; scale bar = 50 µm. Inset, 200×; scale bar = 20 µm.

A nuclear p63 positivity was mostly restricted to the basal layer lining the cystic lesion (Figure 2E). Mesenchymal markers (Vim and αSMA) revealed fine connective tissue and vascular networks (Figure 2F). The Ki67 and PCNA nuclear expressions of the epithelial and lymphoid components were low (29% and 54% positive cells, respectively) (Figure 2G,H). The majority of the lymphoid component of the cyst was CD3-positive. CD3-positive T cells were mainly found in the peripheral lymphoid region. In turn, CD79-positive B cells were mainly identified in the marginal zone. Most lymphocytes located within the epithelium of the cyst were T cells, with occasional B cells. Lymphoid follicles contained scattered CD68-positive macrophages. Several Bcl-2-positive lymphocytes were found in the peripheral lymphoid region and between epithelial cells (the intraepithelial lymphocytes) (Figure 2I–L). The immunoexpression of the CAEV protein was identified in the cells as morphologically consistent with macrophages and macrophage-like cells (most probably dendritic cells) scattered within the lymphoid component (Figure 3A) and, also, at the periphery of the lymphoid region (Figure 3B). The content of the cyst showed no immunopositivity. The presence of the CAEV was also observed in a few epithelial cells of a LEC (Figure 3C) and some ducts of the salivary gland (Figure 3D). Additionally, bronchial epithelial cells and a few macrophages located in the peribronchial interstitial tissue and alveolar lumen were CAEV-positive (Figure 3E,F). No specific labelling was observed in the negative control for CAEV (Figure 3G–I).

## 4. Discussion

The morphological and immunohistochemical features of the cystic lesion that was incidentally found adjacent to the salivary gland in the present goat supported a LEC diagnosis. Our observations were in line with previous human studies that reported LEC as an epithelial-lined cyst in a lymph node adjacent to or embedded in the major salivary gland [3]. Even though a cystic benign lymphoepithelial lesion has been more often found in the parotid glands in HIV-positive patients, there are some reports of a LEC of the submandibular gland also accompanying HIV infection [2,6].

Our goat had a medical history, clinical symptoms, laboratory confirmation, and necropsy diagnosis of CAE. In this animal, respiratory signs due to pneumonia were predominant. Despite the paucity of tissues from this case collected for histopathology, the CAEV infection was identified in the lungs by IHC. Therefore, it seems likely that, as in humans, SRLV-infected cells may migrate into the salivary glands. Additionally, both SRLV and HIV are closely related retroviruses that replicate in monocyte/macrophage cell lineages, affect mucosal membranes, have tropism to biological secretions, and cause latent and chronic multisystemic infections. Contrary to HIV, SRLV does not replicate in CD4^+^ T lymphocytes and is unable to cause immunosuppression in infected goats [14]. Although the main target cells for SRLV are macrophages, monocytes, and dendritic cells, many studies have reported that it could infect a broad range of cells, such as epithelial cells, oligodendrocytes, astrocytes, endothelial cells, fibroblasts, and adipocytes [14].

In the present case, the immunohistochemical analysis confirmed the presence of CAEV (p28 Gag antigen) in the cells morphologically consistent with macrophages and dendritic cells of a LEC and epithelial cells of the salivary gland. Previous studies identified the CAE viral particles in multiple caprine tissues immunohistologically, e.g., lungs; brain; mammary gland; synovial fluid; bronchoalveolar lavage fluid; colostrum/milk (macrophages, macrophage-like cells, uterine, mammary epithelial cells, and bronchial epithelium); lymph nodes; and spleen (macrophages and dendritic cells) [16].

The immunohistochemical studies on a LEC in HIV-infected patients have revealed HIV-1 p24 in residual dendritic cells of lymphoid follicles (follicular dendritic reticulum cells) and follicular and interfollicular macrophages [9,10,12]. Some authors have shown that the lymphoepithelial cyst contained a large amount of HIV-1 p24 and RNA copies, suggesting this lesion to be a reservoir of HIV [2,12].

To the best of our knowledge, there are no published reports confirming the involvement of bacterial or fungal infections in LEC etiopathogenesis. In our study, these pathogens were not present in the lesion, and bacterial pneumonia occurred as a secondary infection.

The definition and etiopathogenesis of the LEC have not been established yet, and many theories exist in this respect. The term “benign lymphoepithelial cyst” was proposed by Bernier and Bhaskar in 1958 to distinguish this lesion from other cystic lesions derived from embryonic branchial pouch remnants [17]. They emphasized an inclusion theory—according to which, the LEC is not an embryologic remnant. The cyst within lymph nodes associated with salivary glands results from the cystic degeneration of the salivary gland epithelium entrapped in the upper cervical lymph nodes during embryonic life. Recently, some authors have suggested sialadenitis-based pathogenesis (in non-HIV-cases), where the dilatation of a duct within the salivary gland is stimulated by focal sialadenitis. As a consequence, a lymphoepithelial cyst is formed by demarcation from the salivary gland because of the granulation tissue response, and it does not arise from intraparotid lymph nodes [18]. For the present case, the inclusion hypothesis would seem a possible explanation for the LEC, whereas underlying sialadenitis was not observed. Additionally, we assume that the possible association with CAEV infection might be relevant to the development of a LEC. Another concept was that the LEC originated from the epithelial remnants of the cervical sinus or the branchial cleft retained during embryogenesis (classic theories: cervical sinus theory and branchial apparatus theory, respectively); therefore, the name “branchial cleft cyst” was coined [17]. Although the final diagnosis of the lesion is based on the histopathological examination, the ability to establish the true nature of salivary LEC in relation to possible theories of origin remains difficult. To date, there have been no specific markers or features to distinguish them from each other. Despite different terms and theories, the branchial cleft cyst and the lymphoepithelial cyst shared similar microscopic features, thus remaining indistinguishable. On the other hand, due to their rarity and incompletely understood pathogenesis, they are still poorly characterized.

Given the different nature of HIV and CAEV infections, the comparisons regarding the clinical manifestation of the LEC is difficult. It is well-known that, in humans, the LEC diagnosis, especially in the parotid gland, is usually concomitant with the early clinical manifestation of HIV infection. However, in the present goat, the lesion did not cause a noticeable deformation, and it was discovered incidentally during necropsy in an animal manifesting the chronic disease. In turn, our observations might not reflect the real situation because of the lack of animals in the early stage of infection.

The pathophysiology of HIV-associated LEC is still debated. It has been postulated that reactive lymphoid proliferation in the parotid gland is responsible for the ductal obstruction and subsequent cyst formation. The other theory suggests that reactive lymphoid proliferation occurs in the lymph nodes of the parotid gland or submandibular gland. The metaplasia in the salivary ducts is caused by the migration of HIV-infected cells, which indirectly leads to cyst formation [2,3,17].

Based on some studies, the lymphoid component of LECs are not as well-organized as the normal lymph nodes (e.g., cell arrangement, proliferative activity, and lack of sinuses and capsule). It may also be merged into the surrounding lymph nodes and completely replaced. On the other hand, LECs may also occur without the lymph nodes and arise outside the lymph nodes, originating from the intralobular ducts; hence, in such a location, the lymphoid component could occur as a secondary lymphocytic infiltration [6,11,17,18,19]. Other authors concluded that it was impossible to determine if a LEC was derived from lymph nodes or from lymphocytic infiltration [20].

Although HIV shows tropism to the lymphoid tissue and its high concentrations can be found within lymphoid nodes, most LEC cells do not contain viral antigens. The accumulation of the virus was reported in the cystic contents due to the shedding of virus-infected cells from the adjacent lymphoid tissues [12]. Moreover, the regression of HIV-associated LEC after antiviral therapy was observed as a decline in the viral load, suggesting some degree of immune recovery [7,12]. Although the detection of HIV-1 p24 antigen by IHC should be performed on lymphoepithelial lesions in HIV-positive patients with salivary gland swelling, and its presence has been considered supportive evidence for the pathogenetic role in HIV-related LEC, there is no clear consensus on LEC etiopathogenesis. Furthermore, some studies have demonstrated the presence of other viruses within a LEC and showed that LECs share similar features in both HIV-positive and HIV-negative patients [9,10,11,18,19].

Our observation may obviously be coincidental, given the scarcity of published data. Despite the high seroprevalence of CAEV in the herd and goats with clinical, pathological, and histopathological features of CAE, only one animal showed the presence of a LEC. Based on our observations, we can only speculate that this lesion may be very rare, as it has been reported in humans. The incidence of the salivary LEC corresponded to less than 6% of the HIV-infected patients, but the exact incidence of the LEC in non-HIV patients is unknown [2,8]. 

To date, only one study has demonstrated unilateral cysts in the upper neck in Anglo-Nubian goats [21]. The authors suggested that the cysts were derived from the salivary gland ducts. However, a small number of lymphoid cells was noted only in one case. In contrast to our report, these cysts were filled with fluids or mucinous materials and were surrounded by a granulated tissue. 

The histological findings in our case were in accordance with those reported in human medicine. The LEC was predominantly lined by a squamous epithelium surrounded by lymphoid tissue [1,3]. Although the tingible body macrophages, lymphocyte exocytosis, and multinucleated giant cells may be present [18], the latter was not demonstrated in our case. 

The LEC contained eosinophilic materials with foamy macrophages and lymphocytes; in addition, some crystalloids were dispersed. A variety of crystalloids have been reported in normal as well as neoplastic and non-neoplastic disorders of salivary glands; however, they are not common. The determination of crystalloid types may be helpful in differentiating the salivary gland tumours. Amylase crystalloids were reported as nonbirefringent geometrical crystalline structures of variable shapes and sizes (polygonal and plate-like) only in benign lesions, including the lymphoepithelial cyst [22,23]. They stained eosinophilic in HE, blue in MGG, bright orange in Pap, and did not react with mucous stains. It has been suggested that they can result from amylase-saturated saliva due to its stagnation in the cyst [24].

Some studies have shown the immunohistochemical features of LEC to be similar in both HIV-associated and non-HIV-associated patients [18,19]. As far as we know, there are no data on the immunohistochemical analysis of LEC in the veterinary literature. In the present study, most T-positive lymphocytes were concentrated in the peripheral interfollicular region and at sites of the intraepithelial population, which was consistent with previously published reports [7]. On the other hand, some authors have demonstrated that intraepithelial lymphocytes are derived predominantly from the B-cell lineage. The majority of lymphocytes of LEC in the lymphoepithelial islands were B cells in both HIV-associated and non-HIV-associated patients [11,25]. Given the previous reports, LEC showed a low proliferative index [18,19,25]. The Bcl-2-positive lymphocytes were not observed in lymphoid germinal centres; however, they were interspersed throughout the lesion, including the intraepithelial population [7,25]. In human cases, one study demonstrated Bcl-2-positive intraepithelial lymphocytes in non-HIV-associated LEC [25].

## 5. Conclusions

As far as we know, this study is the first to describe the occurrence of a LEC of the salivary gland in a goat. Although particularly rare, this salivary lesion should be differentiated from other non-neoplastic cysts, as well as malignant lesions reported in human and veterinary medicine, e.g., mucocele, lymphoepithelial sialadenitis, lymphoma, adenocarcinoma, and mucoepidermoid carcinoma [23]. Histopathology is a gold standard for a LEC diagnosis. We showed that the LEC in a goat shared similar microscopical features to those reported in humans, and our results link these findings to lentiviral infections. As we made quite similar observations, we believe that—thanks to our study—more attention will be paid to this rare lesion in animals. Although the case provides the first evidence for CAEV-associated LEC, which correlates with the serological test for SRLV in a goat, further studies are needed to determine the underlying etiopathogenesis of this lesion.

## Figures and Tables

**Table 1:** The antibodies used for immunohistochemical analysis.

Antibody	Host	Type	Dilution	Source
CK MNF116	Mouse	Monoclonal	1/50	Dako, Agilent Tech., Santa Clara, CA, USA
CK5/6	Mouse	Monoclonal	1/50	Dako, Agilent Tech., Santa Clara, CA, USA
CK14	Mouse	Monoclonal	1/50	Santa Cruz Biotechnology Inc., Dallas, TX, USA
CK19	Mouse	Monoclonal	1/100	Santa Cruz Biotechnology Inc., Dallas, TX, USA
p63	Mouse	Monoclonal	1/50	Dako, Agilent Tech., Santa Clara, CA, USA
Vim	Mouse	Monoclonal	1/50	Dako, Agilent Tech., Santa Clara, CA, USA
αSMA	Mouse	Monoclonal	1/100	Dako, Agilent Tech., Santa Clara, CA, USA
Ki-67(MIB1)	Mouse	Monoclonal	1/75	Dako, Agilent Tech., Santa Clara, CA, USA
PCNA	Mouse	Monoclonal	1/50	Dako, Agilent Tech., Santa Clara, CA, USA
CD3	Rabbit	Polyclonal	1/50	Dako, Agilent Tech., Santa Clara, CA, USA
CD79α	Mouse	Monoclonal	1/50	Dako, Agilent Tech., Santa Clara, CA, USA
CD68	Mouse	Monoclonal	1/100	Dako, Agilent Tech., Santa Clara, CA, USA
Bcl-2	Mouse	Monoclonal	1/50	Dako, Agilent Tech., Santa Clara, CA, USA
CAEP10A1	Mouse	Monoclonal	1/100	VMRD Inc., Pullman, WA, USA

CK—cytokeratin; Vim—vimentin; αSMA—alpha smooth muscle actin; PCNA—Proliferating Cell Nuclear Antigen.
